# Effect of Dietary Manganese on the Growth Performance, Lipid Metabolism, and Antioxidant Capacity in the Post-Larval Coho Salmon (*Oncorhynchus kisutch*)

**DOI:** 10.3390/ani13081310

**Published:** 2023-04-11

**Authors:** Dongwu Liu, Lingyao Li, Qin Zhang, Hairui Yu

**Affiliations:** 1 School of Life Sciences and Medicine, Shandong University of Technology, Zibo 255049, China; liudongwu@sdut.edu.cn; 2 Weifang Key Laboratory of Coho Salmon Culturing Facility Engineering, Institute of Modern Facility Fisheries, College of Biology and Oceanography, Weifang University, Weifang 261061, China; 3 Guangxi Key Laboratory for Polysaccharide Materials and Modifications, Guangxi Marine Microbial Resources Industrialization Engineering Technology Research Center, School of Marine Science and Biotechnology, Guangxi Minzu University, Nanning 530008, China

**Keywords:** manganese requirement, proximate composition, fatty acid synthetase, mTOR signaling, feeding efficiency

## Abstract

**Simple Summary:**

As one of the essential mineral elements, manganese (Mn) is involved in maintaining the growth and physiological function of fish species. An optimal dietary level of manganese accelerated the growth, lipid metabolism, Mn accumulation, and antioxidant capacity of coho salmon. The dietary Mn requirement for post-larvae coho salmon was 17.35 mg kg^−1^ and 19.75 mg kg^−1^ according to the SGR and FCR, respectively. An optimal dietary level of manganese had a positive effect on the antioxidant capacity by scavenging free radicals in fish bodies. In addition, dietary Mn improved lipid metabolism, and the signaling pathway of PI3K/AKT/mTOR may be involved in regulating the activity of lipid metabolism enzymes.

**Abstract:**

In this study, post-larval coho salmon *Oncorhynchus kisutch* (initial weight 0.37 ± 0.03 g) were fed with 6 experimental diets with increasing manganese (Mn) content (2.4, 8.5, 14.8, 19.8, 24.6, and 33.7 mg kg^−1^) for 12 weeks. Our results indicated that the feed conversion rate (FCR), specific growth rate (SGR), condition factor (CF), crude protein, moisture, crude lipid, ash, whole-body Mn, and vertebral Mn were affected by the elevation of Mn content in the diet. The activities of hepatic GSH-PX, Mn-SOD, and CAT were enhanced with increasing Mn content in the diet and reached the highest value at 19.8 mg kg^−1^ Mn. However, the level of hydrogen peroxide (H_2_O_2_), superoxide anion (O_2_^·−^), and malondialdehyde (MDA) was reduced with increasing Mn content in the diet. In addition, the activity of hepatic lipase (HL) and lipoprotein lipase (LPL) was increased with the elevation of dietary Mn content and reached a peak value at 14.8 mg kg^−1^ Mn. The activity of fatty acid synthetase (FAS) and the content of nonesterified fatty acid (NEFA) were increased following the elevation of Mn content from 2.4 to 19.8 mg kg^−1^ in the diet. The results indicated that the appropriate dietary Mn supplementation improved the feeding efficiency, lipid metabolism, and antioxidant capacity of coho salmon. The dietary Mn requirement for post-larval coho salmon was 17.35 mg kg^−1^ and 19.75 mg kg^−1^ based on the SGR and FCR, respectively. An optimal dietary level of Mn enhances hepatic lipid metabolism, and the signaling pathway of PI3K/AKT/mTOR may be involved in regulating the activity of enzymes related to lipid metabolism.

## 1. Introduction

As one of the essential mineral elements, manganese (Mn) is involved in maintaining the growth, physiological function, development, and larval survival of fish species [1]. In addition, Mn is an essential constituent of anti-oxidant enzymes and participates in regulating the metabolism of fatty acids and amino acids [2]. Previously, it was observed that Mn content in fish tissues is positively correlated with dietary Mn levels [3]. Dietary Mn affects a variety of biochemical and metabolic processes in fish species. In addition, it also enhances the activation of superoxide dismutase (SOD) and innate immunity as an enzyme activator [4,5].

An appropriate amount of exogenous Mn should be added to the diet to promote the growth of the fish. The dietary Mn requirements have been studied in various fish species, such as 2.4 mg kg^−1^ Mn for fingerling channel catfish [3], 7.5–10.5 mg kg^−1^ Mn for Atlantic salmon, 12.0–13.0 mg kg^−1^ Mn for rainbow trout, and 12.7 mg kg^−1^ Mn for juvenile hybrid grouper [6,7]. In addition, the appropriate dietary Mn content is 15 mg kg^−1^ for juvenile grouper and 19–29 mg kg^−1^ for juvenile flounder [8,9].

Both Mn deficiency and Mn overload have negative effects on the growth and development of fish species. The typical symptoms of Mn deficiency in animals include weight gain/loss, skeletal deformity, convulsions, breeding disorders, and movement disorders. The growth of juvenile yellow catfish is inhibited as Mn content drops below 3.1 mg kg^−1^ in the diet [10]. Mn deficiency causes skeletal deformity in juvenile gibel carp if Mn content is lower than 13.03 mg kg^−1^ in the diet [5]. Moreover, Mn deficiency inhibits the feed intake of juvenile grouper if dietary Mn content is lower than 15 mg kg^−1^ in the diet [8]. Similarly, Mn overload is also toxic for fish species. Mn overload results in an imbalance of metal ions in the yearling brook charr [11], and Mn overload affects glucose assimilation in the tilapia [12]. The survival, weight gain, and feed efficiency of aquatic animals are reduced if there is excessive Mn content in the diet [6].

The coho salmon (*Oncorhynchus kisutch*) is mainly distributed in the northern Pacific Ocean. As a highly migratory fish, coho salmon has high salinity adaptability. There are rich nutrients in the muscle of coho salmon, such as various polyunsaturated fatty acids and vitamins [13]. It has been found that an optimal dietary selenium requirement is 0.39–0.43 mg kg^−1^ for coho salmon [14]. The optimal dietary Cu and Fe requirements for post-larval coho salmon are 5.1 mg kg^−1^ and 101.7 mg kg^−1^, respectively, based on the specific growth rate [15,16]. For the crucial role of Mn in fish growth and skeletal development, it is interesting to study trace mineral nutrition in larval and post-larval fish [1]. Moreover, the signaling of PI3K/AKT/mTOR participates in regulating lipid metabolism and antioxidant capacity [17,18,19,20]. In this study, we investigated the appropriate Mn requirement and examined the effect of dietary Mn on growth performance, lipid metabolism, antioxidant capacity, and PI3K/Akt/mTOR signaling in coho salmon.

## 2. Materials and Methods

### 2.1. Diet Formulations

According to the appropriate dietary Mn content for juvenile grouper and juvenile flounder [8,9], the diets were formulated according to the ingredients and amounts listed in Table 1. The protein source was casein and gelatin, and the fat source included fish oil and soybean oil. The different contents of Mn sulfate (MnSO_4_·H_2_O) were added into the basal diet to make 6 experimental diets, and the final Mn content in the diets was 2.4, 8.5, 14.8, 19.8, 24.6, and 33.7 mg kg^−1^ Mn, which was analyzed by an inductively coupled plasma atomic emission spectrometer (ICP-AES, Agilent 5100).

### 2.2. Feeding Trial

The post-larvae of coho salmon were obtained from one of the hatcheries in Linyi City (Shandong Province, China). Before starting the formal experiment, salmons (initial weight 0.37 ± 0.03 g) were reared in the plastic tanks (4 × 4 × 4 m) for 2 weeks to acclimate to the culture conditions. Then, the fish were starved for 24 h and weighed. A total of 1800 fish were randomly put into 18 plastic tanks (240 L, 80 × 60 × 60 cm, L × W × H). Each diet was fed to coho salmon in three tanks. Fish were fed 4 times (7:30, 10:00, 14:30, and 17:00) per day for 12 weeks. Fish were cultured in ambient photoperiod (12L:12D), and water temperature, pH, and dissolved oxygen were 15.5 ± 0.5 °C, 6.9 ± 0.3, and 9.5 ± 0.8 mg L^−1^, respectively.

### 2.3. Sampling Procedures

12 weeks later, the fish were starved for 24 h and anesthetized with 150 mg L^−1^ MS222. Then, the total weight of fish in each tank was weighted to determine the condition factor (CF), specific growth rate (SGR), and feed conversion rate (FCR). Then, 10 fish were randomly sampled for analyzing the whole body and vertebral Mn content. Another 10 fish were sampled from each tank and stored at −20 °C for body composition analysis. Moreover, the livers of 10 fish were collected for biochemical and molecular analysis.

### 2.4. Whole-Body Composition Analysis

The moisture, ash, crude protein, and ether extract content in diets were detected by a previous method [21]. The Kjeldahl procedure method was used for analyzing crude protein levels, and water content in samples was measured by drying at 105 °C. The ash content was detected in a muffle furnace by combustion at 550 °C for 24 h. The Soxhlet method was used to detect the content of crude lipids. The Mn concentrations in various samples were detected by an ICP-AES (Agilent 5100).

### 2.5. Hepatic Enzyme Activity Analysis

The liver samples were homogenized in 0.1 mol L^−1^ PBS (pH 7.4) at 4 °C, and the supernatants were collected after 10 min centrifugation at 4 °C. The commercial kits were used to assay the values of malondialdehyde (MDA), glutathione peroxidase (GSH-PX), Mn-superoxide dismutase (Mn-SOD), catalase (CAT), hydrogen peroxide (H_2_O_2_), superoxide anion (O_2_^·−^), and protein concentration. The activities of hepatic lipase (HL), fatty acid synthetase (FAS), lipoprotein lipase (LPL), and the content of nonesterified fatty acid (NEFA) were analyzed by using commercial kits. All commercial kits were purchased from the Nanjing Jiancheng Bioengineering Institute (Nanjing, China).

### 2.6. Real-Time Quantitative Polymerase Chain Reaction

The primer sequences for target genes and reference gene EF1α were listed in Table 2. Real-time PCR was performed by using a quantitative thermal cycler (Roche, Lightcycler96, Switzerland) with the SYBR^®^ Premix Ex Taq™ II (Takara, Japan). The real-time PCR program was as follows: 50 °C for 2 min, 95 °C for 10 min, 40 cycles of 95 °C for 15 s, and 60 °C for 1 min. The 2^−ΔΔCT^ method was used to analyze the gene expression levels.

### 2.7. Calculations and Statistical Analysis

The values were calculated with the following formulae:

SGR (specific growth rate, %/d) = 100 × [Ln (final body weight) − Ln (initial body weight)]/days

HI (hepatic index, %) = 100 × (liver weight/body weight)

CF (condition factor, %) = 100 × (body weight/body length^3^)

VSI (viscerosomatic index, %) =100 × (viscera weight/body weight)

PER (protein efficiency ratio, %) = 100 × (final body weight − initial body weight)/feed intake × dietary protein content

FCR (feed conversion rate) = feed intake/(final body weight − initial body weight)

The statistical analyses were performed with the SPSS 25.0 software. Firstly, homoscedasticity (Levene’s test) and normality (Shapiro–Wilk test) assumptions were made, and one-way analysis of variance (one-way ANOVA) followed by Tukey HSD was used to determine whether various Mn levels significantly (*p* < 0.05) affected the observed responses. Then, a follow-up trend analysis was performed using orthogonal polynomial contrasts to determine whether the significant effects were linear and/or quadratic. All data were expressed as mean ± SE. In addition, a two-tailed Pearson Correlation test was used to determine the correlation between the dietary Mn levels and the values of antioxidant capacity, lipid metabolism enzymes, and gene expression levels. Based on the SGR and FCR, an optimal dietary Mn requirement was estimated using the quadratic regression model.

## 3. Results

### 3.1. Effect of Dietary Mn on Feed Utilization and Growth Performance

**Figure 1:**
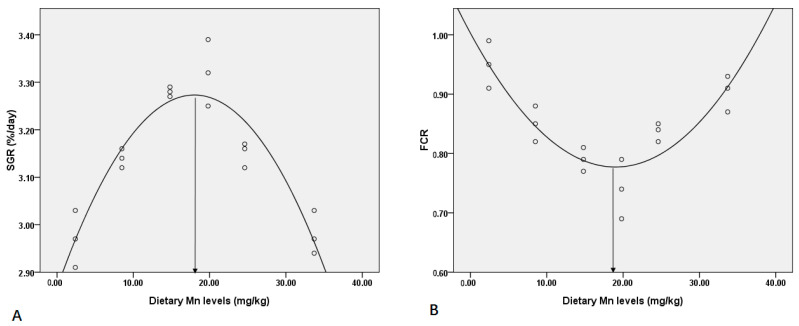
Quadratic regression analysis of SGR and FCR with increasing dietary Mn fed to coho salmon for 12 weeks. (**A**) Dietary Mn requirement was 17.35 mg kg^−1^ according to SGR; (**B**) Dietary Mn requirement was 19.75 mg kg^−1^ according to FCR.

The effect of dietary Mn on growth performance and feeding efficiency of post-larval coho salmon is shown in Table 3. Various dietary Mn levels had no significant influence on the CF, HI, and VSI (Table 3). In addition, CF demonstrated a significant linear trend (*p* < 0.05), but HI demonstrated a significant quadratic effect (*p* < 0.05) (Table 3). However, no significant linear or quadratic trend was observed on the VSI (*p* > 0.05) (Table 3). During the 12-week feeding trial, dietary Mn induced a significant effect on SGR, FCR, and PER of post-larval coho salmon (*p* < 0.05) (Table 3). With the increase in dietary Mn concentration, SGR peaked at 19.8 mg kg^−1^ Mn (Table 3). The highest FCR was at 2.4 mg kg^−1^ Mn, and the lowest FCR was at 19.8 mg kg^−1^ Mn (Table 3). Furthermore, PER demonstrated a significant linear trend (*p* < 0.05), but SGR and FCR showed a significant quadratic effect (*p* < 0.05) (Table 3). A quadratic regression analysis was conducted to illustrate the relationship of SGR and FCR with increasing dietary Mn. The dietary Mn requirement for post-larval coho salmon was 17.35 mg kg^−1^ and 19.75 mg kg^−1^ based on SGR and FCR, respectively (Figure 1).

### 3.2. Effect of Dietary Mn on the Whole-Body Biochemical Composition

The effect of dietary Mn on the indexes of whole-body biochemical components is shown in Table 4. Dietary Mn had a significant effect on the crude protein, moisture, crude lipid, and ash of coho salmon (Table 4). The 24.6 and 33.7 mg kg^−1^ Mn diets significantly enhanced the content of crude protein and crude lipid compared to the other Mn diets (*p* < 0.05) (Table 4). In addition, crude lipid demonstrated a significant linear trend (*p* < 0.05), but crude protein demonstrated a significant linear and quadratic effect (*p* < 0.05) (Table 4). Nevertheless, the moisture and ash displayed a trend of decrease with increasing Mn content in the diets (Table 4). A significant linear and quadratic effect (*p* < 0.05) was also found on the moisture and ash of fish (Table 4).

### 3.3. Effect of Dietary Mn on the Vertebral and Whole-Body Mn Content

Mn content in the whole body and vertebrae was significantly affected (*p* < 0.05) by various Mn diets (Table 5). The whole-body Mn deposition was increased with the elevation of dietary Mn and reached the maximum at 24.6 mg kg^−1^ Mn (Table 5). In addition, the vertebral Mn deposition increased with the elevation of dietary Mn and reached the highest value at 19.8 mg kg^−1^ Mn (Table 5). Both whole-body and vertebral Mn contents showed a significant (*p* < 0.05) linear and quadratic effect (Table 5).

### 3.4. Effect of Dietary Mn on the Activities of Antioxidant Enzymes in the Liver

**Figure 2:**
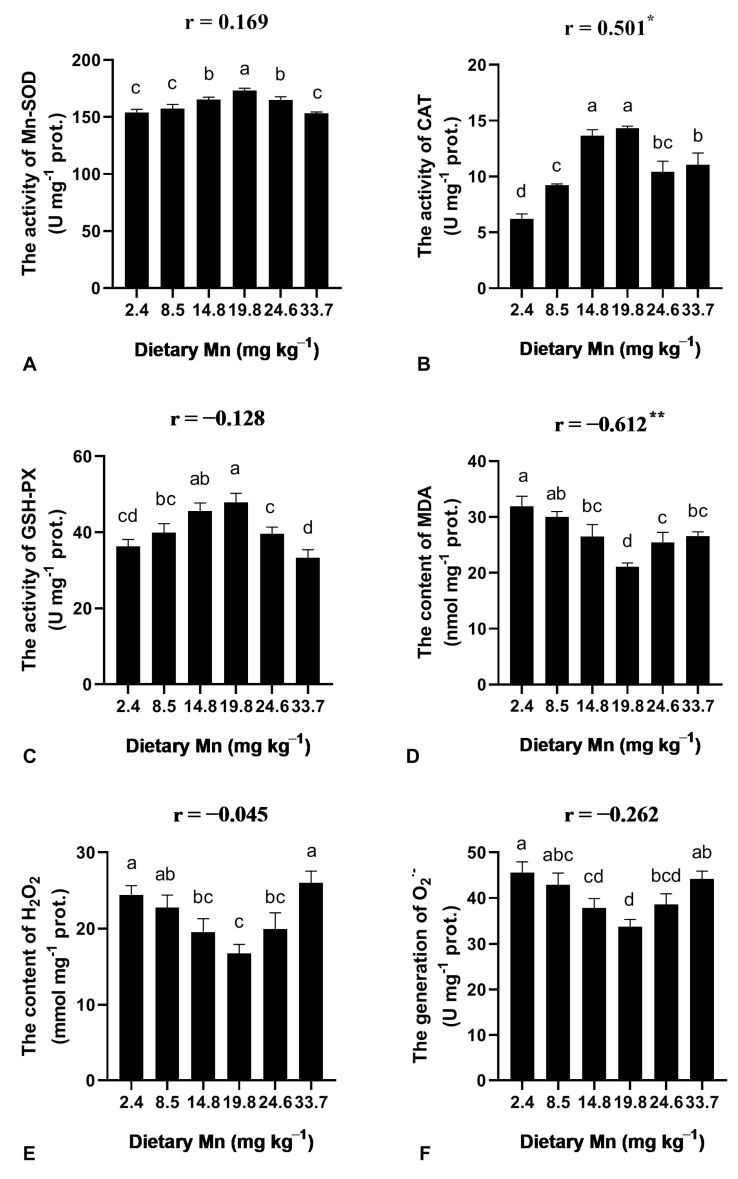
Effect of dietary Mn on the activities of antioxidant enzymes and ROS levels in the liver of post-larval coho salmon. (**A**) Mn-SOD activity; (**B**) CAT activity; (**C**) GSH-PX activity; (**D**) MDA content; (**E**) H_2_O_2_ content; (**F**) Generation O_2_^·−^. Data were expressed by mean ± SE (*n* = 3). One-way ANOVA followed by Tukey HSD was used to determine whether Mn levels significantly (*p* < 0.05) affected the observed responses. The different superscript letters are significantly different (*p* < 0.05). In addition, a two-tailed Pearson Correlation test was used to determine the correlation between dietary Mn levels and the values of antioxidant capacity. One asterisk (*) indicates *p* < 0.05 and two asterisks (**) indicate *p* < 0.01. Abbreviations: malondialdehyde (MDA), glutathione peroxidase (GSH-PX), Mn-superoxide dismutase (Mn-SOD), catalase (CAT), hydrogen peroxide (H_2_O_2_), superoxide anion (O_2_^·−^).

The effect of dietary Mn on the activities of antioxidant enzymes is shown in Figure 2. The activities of Mn-SOD, CAT, and GSH-PX were enhanced as Mn increased from 2.4 to 19.8 mg kg^−1^ Mn and reached the maximum value at 19.8 mg kg^−1^ Mn (Figure 2). Nevertheless, the levels of MDA, H_2_O_2_, and O_2_^·−^ steadily decreased as Mn increased from 2.4 to 19.8 mg kg^−1^ (Figure 2). A significant correlation was observed between dietary Mn levels and the values of CAT activity and MDA content (Figure 2).

### 3.5. Effect of Dietary Mn on the Activities of Lipid Metabolism Enzymes in the Liver

**Figure 3:**
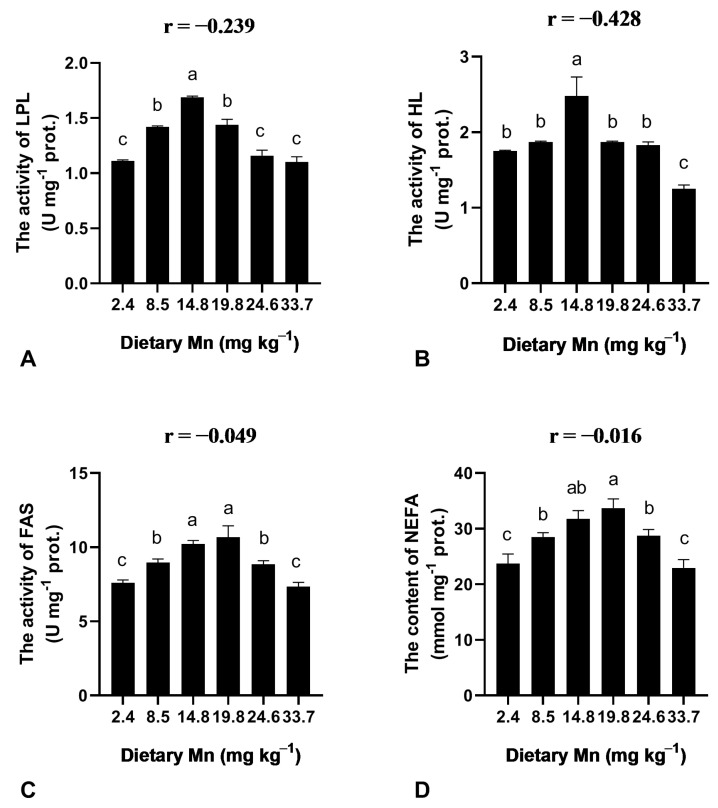
Effect of dietary Mn on the activities of lipid metabolism enzymes and NEFA content in the liver of post-larval coho salmon. (**A**) LPL activity; (**B**) HL activity; (**C**) FAS activity; (**D**) NEFA content. Data were expressed by mean ± SE (*n* = 3). One-way ANOVA followed by Tukey HSD was used to determine whether Mn levels significantly (*p* < 0.05) affected the observed responses. The different superscript letters are significantly different (*p* < 0.05). In addition, a two-tailed Pearson Correlation test was used to determine the correlation between dietary Mn levels and the values of lipid metabolism. Abbreviations: lipoprotein lipase (LPL), hepatic lipase (HL), fatty acid synthetase (FAS), nonesterified fatty acid (NEFA).

The activities of LPL and HL were increased with the elevation of Mn content from 2.4 to 14.8 mg kg^−1^ in the diets and then decreased as Mn increased from 19.8 to 33.7 mg kg^−1^ Mn (Figure 3). The concentrations of 8.5, 14.8, and 19.8 mg kg^−1^ Mn induced a significant (*p* < 0.05) increase in LPL activity, and the highest LPL activity was observed at 14.8 mg kg^−1^ Mn (Figure 3). In addition, the activity of FAS and the content of NEFA significantly (*p* < 0.05) increased as Mn increased from 2.4 to 19.8 mg kg^−1^ (Figure 3).

### 3.6. Effect of Dietary Mn on the Gene Expression Level in the Liver

**Figure 4:**
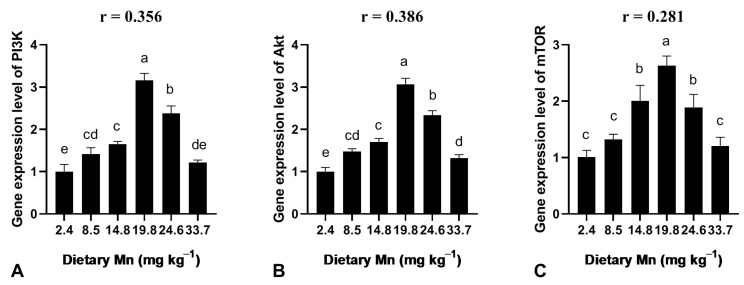
Effect of dietary Mn on the gene expression level in the liver of post-larval coho salmon. (**A**) PI3K; (**B**) Akt; (**C**) mTOR. Data were expressed by mean ± SE (*n* = 3). One-way ANOVA followed by Tukey HSD was used to determine whether Mn levels significantly (*p* < 0.05) affected the observed responses. The different superscript letters are significantly different (*p* < 0.05). In addition, a two-tailed Pearson Correlation test was used to determine the correlation between dietary Mn levels and the values of gene expression levels. Abbreviations: phosphatidylinositol 3-Kinase (PI3K), protein kinase B (Akt), mammalian target of rapamycin (mTOR).

The gene expression level of PI3K, AKT, and mTOR in the liver of post-larval coho salmon is demonstrated in Figure 4. The gene expression level of PI3K, AKT, and mTOR was significantly (*p* < 0.05) increased following the elevation of Mn content from 2.4 to 19.8 mg kg^−1^ in the diet (Figure 4). The highest values of PI3K, AKT, and mTOR were observed at 19.8 mg kg^−1^ Mn (Figure 4).

## 4. Discussion

In this study, dietary Mn significantly affected the growth performance and feed assimilation efficiency of coho salmon. In the previous study, the optimal supplementation of Mn significantly affected the growth of grass carp [22]. Here, we also observed that the optimal supplementation of dietary Mn induced a significant increase in crude lipid and crude protein in coho salmon. In addition, the lowest FCR and highest SGR were observed at 19.8 mg kg^−1^ Mn. Previously, fish growth was enhanced by an optimal Mn content in the diet, and Mn promotes feed assimilation and protein utilization [22]. Our results also indicate that dietary Mn is indispensable for fish growth, and Mn deficiency may hamper the growth of coho salmon.

In addition, the regression analysis was conducted to illustrate the Mn requirements of coho salmon. It showed that the dietary Mn requirements for post-larval coho salmon were 17.35 mg kg^−1^ and 19.75 mg kg^−1^ Mn based on SGR and FCR, respectively. Previously, it was found that the deficiency of Mn in the diet caused low SGR in juvenile grass carp [6,22]. Similar results on the Mn-deficient diet have been observed in some other fish species, such as channel catfish, rainbow trout, common carp, and juvenile cobia [8,23]. Moreover, the low utilization efficiency of the Mn-deficient diet was also observed in juvenile yellow catfish [23], which was consistent with our results. Previously, the Mn requirement has been found in some other fish species, such as channel catfish (2.4 mg kg^−1^), rainbow trout (12.0–13.0 mg kg^−1^), grass carp (15.0 mg kg^−1^), Atlantic salmon (7.5–10.5 mg kg^−1^), hybrid tilapia (7.0 mg kg^−1^), and juvenile cobia (21.7–24.9 mg kg^−1^) [6,24]. The difference in Mn requirements for various fish species may be because different fish species require different Mn content for growth. In addition, we found that the content of ash gradually decreased with the increasing dietary Mn content. It suggests that the deposition efficiency of mineral elements may be inhibited by higher Mn content in the diets.

Previously, an optimal dietary Mn had a higher CF in Atlantic salmon [25]. However, the results of some other studies suggest that the lower dietary Mn causes higher CF in fish species [5,8,23]. It is known that CF is usually treated as an indicator of fish physique [23,26]. The fish physique can be treated as one of the Mn-deficient signals as it is closely related to the development malformation of the fish skeleton. Here, we found that the higher dietary Mn significantly affected CF in coho salmon. A similar effect of dietary Mn on CF has been found in rainbow trout and gibel carp [5,25].

In the previous studies, it was shown that bone and whole-body Mn content serve as Mn requirement indicators in Atlantic salmon, rainbow trout, and gibel carp [5,25,27]. In juvenile grouper and channel catfish, the content of bone Mn showed a trend of linear increase following the elevation of dietary Mn [3,8]. However, the whole-body and vertebral Mn were positively related to dietary Mn in a certain range of Mn concentrations in Atlantic salmon and gibel carp [5,25]. In this study, we observed that Mn deposition was increased with the elevation of dietary Mn concentration and that the content of vertebral Mn was higher than that in the whole body of the fish. It is known that Mn is essential for the development of fish [28]. The juvenile grass carp needs an optimal dietary Mn content for skeleton development [6,22]. In this study, the relationship between fish growth and dietary Mn levels showed that an optimal Mn deposition could induce the growth of coho salmon.

Moreover, Mn is an enzyme activator and an ingredient of metalloenzymes [29]. There are abundant unsaturated fatty acids in the fish body, and unsaturated fatty acids are easily oxygenized [30,31]. The amount of MDA is usually treated as an indicator of lipid peroxidation in fish tissues. It has been found that higher lipid peroxidation occurs in Mn-deficient rats [32]. However, optimal Mn concentration in the diet reduced oxidative damage in the fish [22]. Our results showed that MDA content decreased with the increase in dietary Mn levels and reached the minimum value at an optimal Mn concentration, which was consistent with the previous findings [22,29]. Thus, optimal dietary Mn can avoid the oxidation hazard to lipids in fish bodies.

The ability to remove free radicals is associated with the activity of antioxidative enzymes in animals [33]. In a previous study, the antioxidative capacity was enhanced by various dietary Mn levels and Mn increased the content of non-enzymatic antioxidants and the activity of antioxidant enzymes [22]. SOD is a crucial endogenous antioxidant enzyme, which scavenges O_2_^·−^ into H_2_O_2_ [34,35]. Our results illustrated that the optimal dietary Mn induced a higher Mn-SOD activity. However, the activity of Mn-SOD in the liver was decreased in fish fed with the Mn-deficient diet, suggesting that the low level of dietary Mn had a lower removal ability of free oxygen. A similar effect of dietary Mn on SOD was found in the Atlantic salmon [25]. In addition, CAT is crucial for scavenging •OH and H_2_O_2_ [34]. It is confirmed that optimal Mn supplementation improved the activities of CAT in the liver and intestine [22]. Here, we found that dietary Mn affected CAT and GSH-PX activities, and the data were consistent with previous studies. Moreover, the optimal dietary Mn reduced the levels of H_2_O_2_ and O_2_^·−^ in coho salmon. It further indicated that the optimal dietary Mn had a positive effect on the antioxidant capacity of coho salmon.

The activity of HL and LPL affects lipid metabolism in animals [36]. We observed that the optimal dietary Mn significantly enhanced LPL and HL activity in coho salmon. Tang et al. observed that lipase activities were elevated with optimal Mn supplementation [22]. We also found that optimal dietary Mn improved lipid metabolism by enhancing the activity of LPL and HL. In addition, we observed that the hepatic FAS activity and NEFA content were significantly enhanced with the elevation of dietary Mn content. Thus, our results confirmed that optimal dietary Mn promotes the activity of enzymes related to lipid metabolism.

The PI3K/AKT/mTOR signaling pathway regulates various cellular processes, including proliferation, survival, metabolism, growth, and metastasis angiogenesis [37]. It is known that AKT is a crucial messenger in the signaling of mTOR. The activated AKT transfers into other cell compartments to stimulate the downstream substrate mTOR [38]. Here, we observed that various dietary Mn levels significantly induced the gene expression level of PI3K. The gene expression levels of AKT and mTOR were also enhanced by various dietary Mn levels. Thus, the nutritional element Mn could induce mTOR signaling by inducing the expression of PI3K and AKT in the liver of the post-larvae of coho salmon. Previously, it has been observed that the signaling of PI3K/AKT/mTOR is involved in regulating lipid metabolism and antioxidant capacity [17,18,19]. Therefore, the signaling pathway of PI3K/AKT/mTOR activated by dietary Mn may be involved in regulating the activity of enzymes related to lipid metabolism and antioxidant capacity.

## 5. Conclusions

An optimal dietary level of Mn accelerated growth, assimilation abilities, Mn accumulation, and antioxidant capacity in the post-larvae of coho salmon. The dietary Mn requirement for post-larval coho salmon was 17.35 mg kg^−1^ and 19.75 mg kg^−1^ according to the SGR and FCR, respectively. Dietary Mn can increase the antioxidant capacity by scavenging free radicals in fish bodies. In addition, dietary Mn enhanced lipid metabolism, and the signaling pathway of PI3K/AKT/mTOR may be involved in regulating the activity of enzymes related to lipid metabolism.

## Figures and Tables

**Table 1:** Formulation and proximate composition of diets for post-larval coho salmon *Oncorhynchus kisutch* (g kg^−1^ dry matter).

Ingredients (g)	Dietary Mn Levels (mg kg^−1^)					
	2.4	8.5	14.8	19.8	24.6	33.7
Casein ^1^	400.0	400.0	400.0	400.0	400.0	400.0
Gelatine ^1^	100.0	100.0	100.0	100.0	100.0	100.0
Dextrin ^1^	160.0	160.0	160.0	160.0	160.0	160.0
α-Cellulose ^1^	95.0	95.0	95.0	95.0	95.0	95.0
Fish oil ^1^	75.0	75.0	75.0	75.0	75.0	75.0
Soybean oil ^1^	75.0	75.0	75.0	75.0	75.0	75.0
Mineral premix, manganese-free ^2^	60.0	60.0	60.0	60.0	60.0	60.0
Vitamin premix ^3^	10.0	10.0	10.0	10.0	10.0	10.0
L-Arg	10.0	10.0	10.0	10.0	10.0	10.0
Ethoxyquin	6.0	6.0	6.0	6.0	6.0	6.0
DL-Met	5.0	5.0	5.0	5.0	5.0	5.0
Choline chloride	3.0	3.0	3.0	3.0	3.0	3.0
Ascorbic acid phosphate	0.5	0.5	0.5	0.5	0.5	0.5
Glycine betaine	0.5	0.5	0.5	0.5	0.5	0.5
Manganese sulfate (mg kg^−1^)	0.0	43.0	55.3	61.5	73.7	86.0
Proximate composition						
Crude protein (%)	42.1	42.08	42.04	42.06	42.03	42.05
Crude lipid (%)	12.57	12.53	12.55	12.57	12.59	12.51
Ash (%)	6.12	6.18	6.13	6.17	6.15	6.19
Moisture (%)	7.3	7.31	7.29	7.32	7.28	7.33
Mn (mg kg^−1^)	2.4	8.5	14.8	19.8	24.6	33.7

^1^ Provided by Shandong Conqueren Marine Technology Co., Ltd., Weifang, China. ^2^ Composition (mg kg^−1^ mineral premix): AlK(SO_4_)_2_·12H_2_O, 124.00; CaCl_2,_ 17880.00; CoCl_2_·6H_2_O, 49.00; FeSO_4_·7H_2_O, 707.00; CuSO_4_∙5H_2_O, 32.00; KCl, 1192.00; KI, 5.00; MgSO_4_·7H_2_O, 4317.00; NaCl, 4934.00; Na_2_SeO_3_·H_2_O, 3.00; ZnSO_4_·7H_2_O, 177.00; Ca(H_2_PO_4_)_2_·H_2_O, 12457.00; KH_2_PO_4_, 9930.00. ^3^ Composition (IU or mg kg^−1^ vitamin premix): retinal palmitate, 10,000 IU; cholecalciferol, 4000 IU; *α*-tocopherol, 75.00 IU; menadione, 22.00 mg; thiamine-HCl, 40.00 mg; riboflavin, 30.00 mg; D-calcium pantothenate, 150.00 mg; pyridoxine-HCl, 20.00 mg; meso-inositol, 500.00 mg; D-biotin, 1.00 mg; folic acid, 15.00 mg; ascorbic acid, 200.00 mg; niacin, 300.00 mg; cyanocobalamin, 0.30 mg.

**Table 2:** Real-time quantitative PCR primers for target and reference genes.

Target Gene	Forward (5′–3′)	Reverse (5′–3′)	GenBank
PI3K	CCAGTGGCTCAAGGACAAGAACAG	GGATGAAGGTGGCTACGCAGTATC	XM_020466892.1
AKT	GAGTTCACGGCACAGACCATCAC	CGTATGCTGGCGGAGTAAGAGAAC	XM_020503531.1
mTOR	GCAACAGCGACAGCGAGGTAG	TGGAGAGGGAGATTGAGCGGAAG	XM_020506200.1
EF1α	ACCGGCCATCTGATCTACAAATGC	CTCACGCTCAGCCTTCAGCTT	XM_031793751.1

**Table 3:** The growth performance and feed utilization of post-larval coho salmon fed a diet with different levels of manganese.

Dietary Mn (mg kg^−1^)	SGR (% Day^−1^)	CF (%)	HI (%)	VSI (%)	FCR	PER
2.4	2.97 ± 0.06 ^c^	0.95 ± 0.05 ^b^	1.86 ± 0.05 ^a^	1.43 ± 0.07 ^a^	0.95 ± 0.04 ^a^	0.025 ± 0.001 ^d^
8.5	3.14 ± 0.02 ^b^	1.02 ± 0.02 ^ab^	1.66 ± 0.02 ^a^	1.48 ± 0.03 ^a^	0.85 ± 0.03 ^bc^	0.029 ± 0.001 ^cd^
14.8	3.28 ± 0.01 ^a^	1.02 ± 0.04 ^ab^	1.64 ± 0.15 ^a^	1.49 ± 0.38 ^a^	0.79 ± 0.02 ^cd^	0.031 ± 0.001 ^bc^
19.8	3.32 ± 0.07 ^a^	1.08 ± 0.08 ^ab^	1.56 ± 0.01 ^a^	1.49 ± 0.05 ^a^	0.74 ± 0.05 ^d^	0.034±0.001 ^ab^
24.6	3.15 ± 0.03 ^b^	1.11 ± 0.17 ^ab^	1.52 ± 0.28 ^a^	1.47 ± 0.39 ^a^	0.84 ± 0.02 ^bc^	0.034 ± 0.003 ^ab^
33.7	2.98 ± 0.05 ^c^	1.27 ± 0.15 ^a^	1.71 ± 0.05 ^a^	1.34 ± 0.15 ^a^	0.90 ± 0.03 ^ab^	0.037 ± 0.002 ^a^
ANOVA *p*-value	0.000	0.034	0.094	0.962	0.000	0.000
Linear trend	0.586	0.002	0.076	0.678	0.066	0.000
Quadratic trend	0.000	0.311	0.021	0.425	0.000	0.170

Data were expressed by mean ± SE (*n* = 3). One-way ANOVA followed by Tukey HSD was used to determine whether Mn levels significantly (*p* < 0.05) affected the observed responses. The means in the same column with different superscript letters are significantly different (*p* < 0.05). In addition, a follow-up trend analysis was performed using orthogonal polynomial contrasts to determine whether the significant effects were linear and/or quadratic. Abbreviations: specific growth rate (SGR), Condition factor (CF), hepatic index (HI), viscerosomatic index (VSI), feed conversion rate (FCR), protein efficiency ratio (PER).

**Table 4:** Whole-body composition of post-larval coho salmon fed a diet with different levels of manganese.

Dietary Mn Levels (mg kg^−1^)	Moisture (%)	Crude Protein (%)	Crude Lipid (%)	Ash (%)
2.4	75.24 ± 0.02 ^a^	12.00 ± 0.84 ^b^	8.45 ± 0.44 ^cd^	3.49 ± 0.03 ^a^
8.5	74.97 ± 0.03 ^a^	12.04 ± 0.08 ^b^	8.22 ± 0.01 ^d^	3.42 ± 0.01 ^ab^
14.8	74.60 ± 0.20 ^ab^	12.37 ± 0.42 ^b^	8.93 ± 0.05 ^bc^	3.37 ± 0.02 ^b^
19.8	74.20 ± 0.07 ^b^	12.23 ± 0.74 ^b^	9.43 ± 0.15 ^ab^	3.20 ± 0.04 ^c^
24.6	72.17 ± 0.08 ^c^	14.20 ± 0.01 ^a^	9.85 ± 0.19 ^a^	3.20 ± 0.04 ^c^
33.7	71.96 ± 0.54 ^c^	14.41 ± 0.28 ^a^	9.94 ± 0.15 ^a^	3.18 ± 0.01 ^c^
ANOVA *p*-value	0.000	0.000	0.000	0.000
Linear trend	0.000	0.000	0.000	0.000
Quadratic trend	0.000	0.016	0.706	0.014

Data were expressed by mean ± SE (*n* = 3). One-way ANOVA followed by Tukey HSD was used to determine whether Mn levels significantly (*p* < 0.05) affected the observed responses. The means in the same column with different superscript letters are significantly different (*p* < 0.05). In addition, a follow-up trend analysis was performed using orthogonal polynomial contrasts to determine whether the significant effects were linear and/or quadratic.

**Table 5:** The whole-body and vertebral Mn content in the post-larval coho salmon fed a diet with different levels of manganese.

Dietary Mn Levels (mg kg^−1^)	Whole-Body Mn Concentration (mg kg^−1^)	Vertebral Mn Concentration (mg kg^−1^)
2.4	1.23 ± 0.01 ^e^	10.56 ± 0.17 ^d^
8.5	2.59 ± 0.04 ^d^	15.63 ± 0.15 ^c^
14.8	3.59 ± 0.04 ^c^	23.12 ± 0.39 ^b^
19.8	3.91 ± 0.01 ^a^	24.78 ± 0.48 ^a^
24.6	3.92 ± 0.04 ^a^	23.79 ± 0.16 ^b^
33.7	3.68 ± 0.03 ^b^	23.68 ± 0.20 ^b^
ANOVA *p*-value	0.000	0.000
Linear trend	0.000	0.000
Quadratic trend	0.000	0.000

Data were expressed by mean ± SE (*n* = 3). One-way ANOVA followed by Tukey HSD was used to determine whether Mn levels significantly (*p* < 0.05) affected the observed responses. The means in the same column with different superscript letters are significantly different (*p* < 0.05). In addition, a follow-up trend analysis was performed using orthogonal polynomial contrasts to determine whether the significant effects were linear and/or quadratic.

## Data Availability

All the data in the article are available from the corresponding author upon reasonable request.
